# Extended reality and computer-based simulation for teaching situational awareness in undergraduate health professions education: a scoping review

**DOI:** 10.1186/s41077-025-00343-5

**Published:** 2025-04-02

**Authors:** Mehak Chandanani, Anita Laidlaw, Craig Brown

**Affiliations:** Institute of Education in Healthcare and Medical Sciences, University of Aberdeen, Aberdeen, UK

**Keywords:** Virtual reality, Extended reality, Scoping review, Situational awareness, Decision-making

## Abstract

**Introduction:**

With the rapid evolution of healthcare environments, effective and accessible experiential learning has become an integral part of health education. Virtual reality (VR) poses the advantage of providing users with a virtual, immersive experience, to allow them to interact with elements of a simulated environment. This scoping review aims to evaluate the use of virtual reality (VR)-based simulation for the training of situational awareness (SA) and decision-making (DM) for undergraduate healthcare education.

**Methods:**

A search was carried out across eight databases, namely: MEDLINE, Embase + Embase Classic, Scopus, Google Scholar, PubMed, CINAHL, ERIC, and PsycINFO. Studies evaluating the use of VR and its extended interfaces (i.e., augmented reality (AR) and mixed reality (MR)) for training SA and DM in undergraduate healthcare education were included.

**Results:**

Of 3932 studies retrieved from the database search, 35 studies were included within the review. VR-based interventions were used across a range of healthcare profession trainees, including nursing, medical, paramedical, midwifery, and healthcare assistant students. Seventeen studies used screen-based VR interventions, and 16 studies used head-mounted devices (HMD). One study used both screen-based and HMD interventions and one further augmented reality. Twenty-nine studies assessed the role of the intervention in DM training, and 6 studies assessed its role in SA training. Eighteen studies used validated assessment tools, and 17 studies used educational theories to underpin their learning techniques.

**Conclusions:**

The role of VR in training of SA and DM for healthcare professions has been well recognized, as demonstrated by the increasing number of studies. There is need for consensus of reporting for such studies to ensure a high-quality body of evidence with standardized outcome assessment.

## Introduction

Continued growing demand for trained healthcare professionals alongside pressured health systems globally has made the need for further development of effective and accessible experiential educational opportunities apparent [1]. Evolving healthcare environments, societal expectations, and rapidly evolving medical research, along with a growing focus on holistic, patient-centred care, has posed significant challenges to traditional healthcare education curricula. The use of technology, including simulation-based methods, in response to these challenges has proved promising for improved knowledge acquisition, decision-making, skill coordination, and preparation for competent practice of healthcare professionals in training [2].

Simulation-based learning is defined as “an array of structured activities that represent actual or potential situations in education and practice” [3]. The concept of simulation can be perceived to be complex, with multiple definitions and categorizations. While simulation has been described as a “technique not a technology” [4], there are several diverse modes of delivery by which simulation-based methods can be classified: human patient actors, mannequin-based simulators, computer-based simulations, or virtual reality simulators (VR), for example [5]. Virtual simulation has been described as a broad term referring to computer-based programs which use varying types of technology to present scenarios; however, previous authors note that terminology in this field is often confusing [6, 7]. Computer-based simulations allow users to interact with patients usually via a screen-based interface [3, 5] which may include a virtual environment as well as virtual patient, whereas VR is a technology which provides the user with an illusion of immersion and presence [7] within a computer-generated environment while allowing them to interact with its elements. Over the past decade, further definitions in the spectrum of VR interfaces have been described with the latest version of the Healthcare Simulation Dictionary [3] including augmented reality (AR), a type of VR where real-world elements are supplemented with computer-generated factors such as audio, graphics used to enhance the learning process [3] and mixed reality (MR), and the merging of virtual and real-world elements along the “virtuality continuum” [3] to facilitate the real-time interaction of digital and physical elements [8]. Often, these technologies are grouped under an umbrella term as extended reality (XR). VR environments have often been described based on the technology being used, for example head-mounted display (HMD-VR) or other technologies such as computer-based virtual reality environments controlled by mouse, keyboard, voice, or haptic devices [8]. More recent descriptions of VR attempt to categorize VR into immersive VR where the user wears a display (such as a HMD) or non-immersive where a combination of screens surrounds the user presenting virtual information [3].

Simulation-based education has been widely used for both practical skills as and non-technical/behavioural skills (NTS) education encompassing the social and cognitive capabilities required within healthcare environments [9]. NTS include but are not limited to decision-making (DM) and situational awareness (SA), which will be the focus of our article. Clinical DM is a complex process which involves combining critical thinking, and clinical knowledge with awareness of the situation, and patient’s wishes [10]. According to the Endsley model, SA is a combination of the perception and comprehension of elements of a situation and surrounding environment, along with the ability to project the future status of the situation [11]. SA is known to contribute to the process of dynamic decision-making, making it a core training skill [11].

While skills of SA and DM are traditionally learnt via experience on clinical placements, research into the development of such skills in an undergraduate population is crucial due to their limited exposure to clinical practice. With increasing numbers of health professions students and the difficulties of assessment of these skills on clinical placements, it is important for clinical educators to adopt new technologies to address this gap [1, 12, 13].

Despite a multitude of review articles examining the use of simulation training for non-technical skills, there seems to be a lack of reviews focussing on the use of VR for the development of SA and DM skills [14–16]. Hence, we sought to examine the use of VR across the spectrum of undergraduate health professionals’ education specifically focussing on the key behavioural skills of SA and DM [17, 18]. In this review, we will use the term “behavioural skills” instead of the more traditional term “non-technical skills” as this better reflects modern understanding of both simulation and real-world practice terminology [17, 18]. Behavioural skills are defined as the cognitive, social, and personal skills that complement technical skills, contributing to safe and efficient task performance [19].

This scoping review aims to evaluate the breadth and depth of the evidence of the use of VR in the teaching and assessment of SA and DM in undergraduate healthcare professions education. Specific questions this review aims to answer are as follows:

1. In what contexts are VR, AR, and MR used within simulation training for SA and DM in healthcare professions education?
2. What outcome measures are used to examine the impacts of VR, AR, and MR use within simulation training for SA and DM?
3. What educational theories underpin VR, AR, and MR use within simulation training in healthcare professions education?

## Methods

The study methodology, rationale, protocol, and search strategy have been previously described [20]. In summary, the scoping review utilized the framework described by Arksey and O’Malley [21]. This consisted of identification of relevant literature, selection of studies, mapping out the data, and synthesizing and reporting of results.

In this review, we used the definition of VR as described by Abbas et al.: “VR is a three-dimensional computer-generated simulated environment, which attempts to replicate real world or imaginary environments and interactions, thereby supporting work, education, recreation, and health” [22]. The principles of a three-dimensional computer-generated simulation of reality to support education were taken to include studies with all extended realities as well as patients represented in virtual environments where participants could interact with both the patient and wider environment including computer-based simulation.

### Databases and search strategy

The scoping review is reported in accordance with the Preferred Reporting Items for Systematic review and Meta-Analysis Scoping Review extension (PRISMA-ScR) (Appendix 2) [23]. A systematic search was conducted to identify relevant studies looking into the use of VR, AR, MR, and computer-based simulation modalities for the training of SA and DM in health professions’ education on 9th July 2023 using Ovid MEDLINE, Embase, PubMed, Google Scholar, CINAHL, Scopus, PsycINFO, and ERIC databases. Relevant synonyms and Medical Subject Headings (MeSH) were used to carry out the search using the keywords as follows: (“Virtual Reality” OR “Mixed Reality” OR “Augmented Reality”) AND “Education” AND “Healthcare Professionals” AND “Non-technical skills”. A detailed description of the search strings used on the databases can be found in Appendix 3.

### Eligibility criteria

Included studies were (1) peer-reviewed empirical primary research studies or published academic work (2) of quantitative or qualitative study designs, (3) looking into VR, AR, MR, or computer-based simulation techniques (4) for the training and assessment of DM and SA (5) in undergraduate healthcare students (6) in clinical workplace or education environments. Studies were excluded from review if as follows: (1) There was no available English language translated text, (2) review articles, and (3) studies which did not include extractable information for outcomes for SA or DM. The reference list for all excluded review articles was screened for texts that met the inclusion criteria.

### Study selection

The results from the database search were screened simultaneously by three independent reviewers (each result being screened by two reviewers) in two stages: (1) title and abstract screening against pre-defined inclusion and exclusion criteria, followed by (2) full-text screening. Reviewers were blinded to each other’s decisions during the screening process. Reviewers were unblinded at the end of each stage, and discrepancies were resolved in the presence of a third adjudicator through discussion and to support calibration for future rounds.

### Data charting

A data extraction form was designed (Appendix 3) to ensure standardized data extraction. The data was extracted under the following headings: Study characteristics (study title, author, journal, year of publication, country of origin, type of study), Participant characteristics (total population selected, number of controls, number of cases), and Outcomes (outcomes measured, mode of measurement, conclusion(s) drawn, educational theories outlined). Relevant data was extracted from all included studies by one reviewer. Where appropriate, data has been displayed in the form of tables supplemented with narrative review.

## Results

### Selection process and study characteristics

**Fig. 1:**
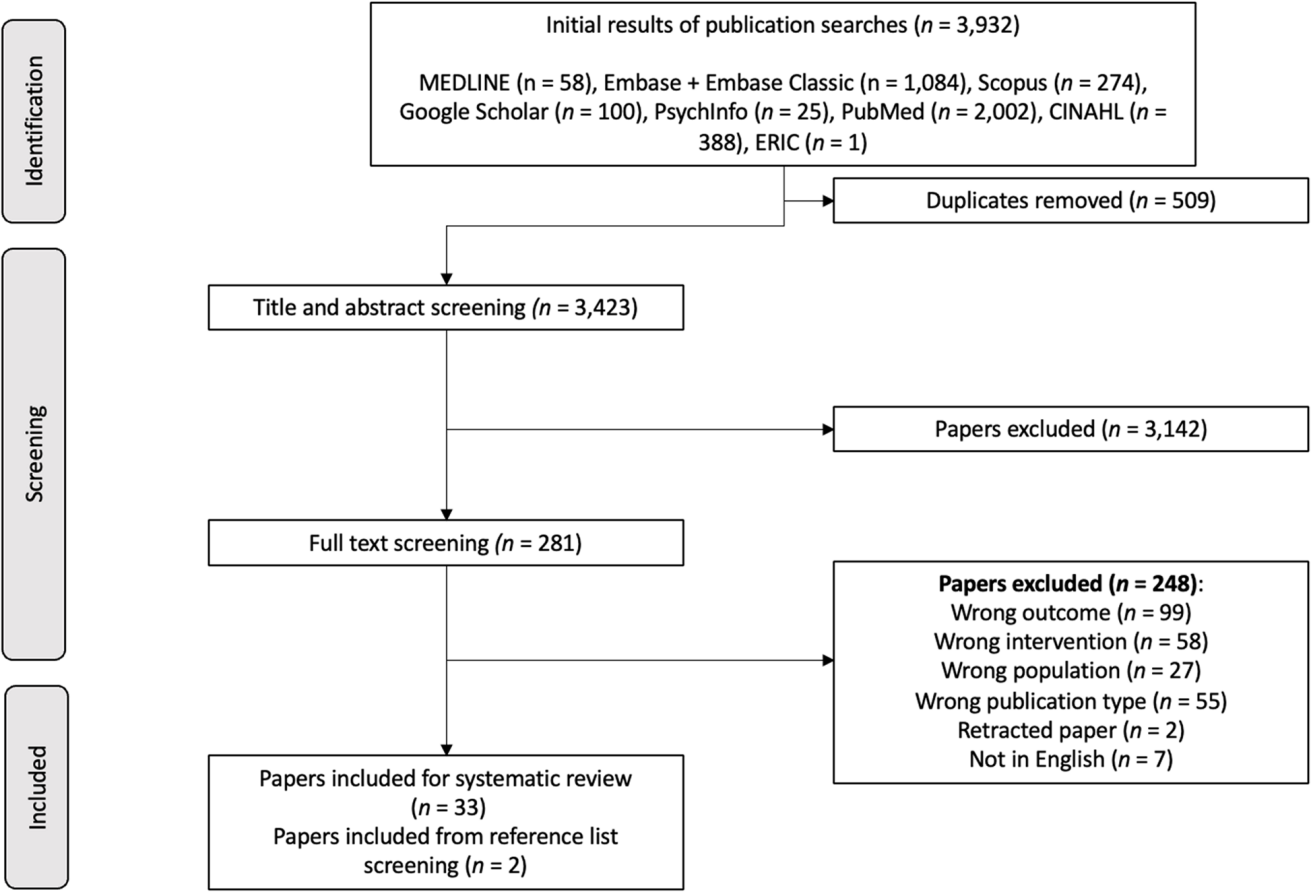
PRISMA flow diagram summary of search and screening process

The process of selection of the included studies has been detailed in Fig. 1. Of 3932 articles retrieved from the database search, 3423 papers were deemed eligible for screening by title and abstract after de-duplication. Two-hundred eighty-one articles were screened by full text for inclusion within the study, of which 33 were included [24–56]. While reviews were excluded from the study, the reference lists of reviews were screened for relevant texts. Two studies were selected for inclusion within the study from reference list screening, resulting in a total of 35 papers deemed eligible for inclusion [57, 58]. Of the 35 studies included for review, 9 studies were from the United States of America (USA) [24, 29, 31, 32, 34, 36, 38, 41, 45], 6 were from South Korea [39, 42, 43, 49, 50, 56], 3 were from the United Kingdom (UK) [47, 57, 58], 3 were from France [26, 27, 46], 2 were from Japan [40, 54], 2 from Australia [48, 51], 2 from Canada [25, 55], 2 from China [33, 35], and 1 each from Switzerland [28], Taiwan [30], Turkey [37], Germany [44], Ireland [52], and Finland [53]. All study characteristics are detailed in Table 1.

**Table 1:** Study characteristics

*Author, year of publication*	Article title	Journal of publication	Country of origin	Type of study
***Adhikari *et al*., 2021 *** [57]	A mixed-methods feasibility study to assess the acceptability of immersive virtual reality sepsis game as an adjunct to nursing education	*Nurse Education Today*	United Kingdom (UK)	Non-randomized pre-post-test feasibility study
***Anbro *et al*., 2020 *** [24]	Using virtual simulations to assess situational awareness and communication in medical and nursing education: a technical feasibility study	*Journal of Organizational Behavior Management*	United States of America (USA)	Pre-post-test comparative feasibility study
***Atthill *et al*., 2021 *** [25]	Exploring the impact of virtual asynchronous debriefing method after a virtual simulation game to support clinical decision-making	*Clinical Simulation in Nursing*	Canada	Pre-post-test comparative study
***Blanie *et al*., 2020 *** [26]	Comparative value of a simulation by gaming and a traditional teaching method to improve clinical reasoning skills necessary to detect patient deterioration: a randomized study in nursing students	*BMC Medical Education*	France	Single-blind randomized controlled study
***Bracq *et al*., 2021 *** [27]	Training situational awareness for scrub nurses: error recognition in a virtual operating room	*Nurse Education in Practice*	France	Pre-post-test study
***Carrard *et al*., 2020 *** [28]	Virtual patient simulation in breaking bad news training for medical students	*Patient Education and Counseling*	Switzerland	Focus-group study
***Casler *et al*., 2022 *** [29]	The effect of asynchronous group discussions on nurse practitioner student debriefing experience in virtual simulation	*Journal of American Association of Nurse Practitioners*	United States of America (USA)	Pre-post-test comparative study
***Chen and Liou, 2022 *** [30]	Development and application of AR-based assessment system for infant airway obstruction first aid training	*Children*	Taiwan	Focus-group qualitative study
***Cieslowski and Haas, 2023 *** [31]	Innovative learning: implementing virtual reality in a large class of prelicensure students using limited equipment, time, and resources	*Nursing Education Perspectives*	United States of America (USA)	Pre-post-test study
***Colonna*** **et al*., 2022*** [32]	Trauma bay virtual reality — a game changer for ATLS instruction and assessment	*Journal of Trauma and Acute Care Surgery*	United States of America (USA)	Single-institution prospective cohort study
***Du *et al*., 2022 *** [33]	A novel scenario-based, mixed-reality platform for training nontechnical skills of battlefield first aid: prospective interventional study	*JMIR Serious Games*	China	Pre-post-test prospective interventional study
***Fogg *et al*., 2020 *** [34]	Using virtual simulation to develop clinical judgement in undergraduate nursing students	*Clinical Simulation in Nursing*	United States of America (USA)	Pre-post-test interventional study
***Hu*** **et al*., 2022*** [35]	Teaching disaster evacuation management education to nursing students using virtual reality mobile game-based learning	*CIN: Computers, Informatics, Nursing*	China, United States of America (USA)	Pre-post-test comparative study
***Jayasundera *et al*., 2022 *** [36]	Virtual reality simulation: evaluating an experiential tool for the clinical application of pathophysiology	*Medical Science Educator*	United States of America (USA)	Pre-post-test interventional study
***Sahin Karaduman and Basak, 2023*** [37]	Is virtual patient simulation superior to human patient simulation: a randomized controlled study	*CIN: Computers, Informatics, Nursing*	Turkey	Randomized controlled trial
***Pardue*** **et al*., 2022*** [38]	Exploring the development of nursing clinical judgement among students using virtual reality simulation	*Nurse Educator*	United States of America (USA)	Interventional study
***Kim*** **et al*., 2023*** [39]	Constructing a mixed simulation with 360° virtual reality and a high-fidelity simulator: usability and feasibility assessment	*CIN: Computers, Informatics, Nursing*	South Korea	Pre-post-test interventional study
***Kiyozumi et al., 2022*** [40]	Development of virtual reality content for learning Japan Prehospital Trauma Evaluation and Care initial assessment procedures	*Acute Medicine and Surgery*	Japan	Prospective cohort study
***Kleinheksel, 2014 *** [41]	Transformative learning through virtual patient simulations: predicting critical student reflections	*Clinical Simulation in Nursing*	United States of America (USA)	Interventional study
***Lee*** **et al*., 2022 *** [42]	Optimizing learning experiences in mental-health nursing education using virtual reality simulation with 360-degree video	*Science, Engineering, and Health Studies*	South Korea	Pre-post-test interventional study
***Lee*** **et al*., 2023 *** [43]	Effectiveness of the patient’s severity classification competency promotion virtual reality program of nursing students during the COVID-19 pandemic period	*Healthcare*	South Korea	Pre-post-quasi-experimental study
***Mahling *et al*., 2023 *** [44]	Virtual reality for emergency medicine training in medical school: prospective, large-cohort implementation study	*Journal of Medical Internet Research*	Germany	Prospective cohort study
**Mascarenhas et al., 2023** [45]	A novel approach to the room of errors (ROE): a three-dimensional virtual tour activity to spotlight patient safety threats	*Cureus*	United States of America (USA)	Prospective cohort study
***McCallum *et al*., 2011 *** [58]	Exploring nursing students’ decision-making skills while in a Second Life clinical simulation laboratory	*Nurse Education Today*	United Kingdom (UK)	Qualitative study
***Michelet *et al*., 2020 *** [46]	Effect of computer debriefing on acquisition and retention of learning after screen-based simulation of neonatal resuscitation: randomized controlled trial	*JMIR Serious Games*	France	Randomized controlled trial
***Middeke *et al*., 2018 *** [47]	Training of clinical reasoning with a serious game versus small-group problem-based learning: a prospective study	*PLOS One*	Germany, United Kingdom (UK)	Prospective cohort study
***Mills*** **et al*., 2020*** [48]	Virtual reality triage training can provide comparable simulation efficacy for paramedicine students compared to live simulation-based scenarios	*Prehospital Emergency Care*	Australia	Prospective cohort study
***Park *et al*., 2022 *** [49]	Learning effects of virtual versus high-fidelity simulations in nursing students: a crossover comparison	*BMC Nursing*	South Korea	Quasi-experimental crossover study
***Rim and Shin, 2022 *** [50]	Development and assessment of a multiuser virtual environment nursing simulation program: a mixed-methods research study	*Clinical Simulation in Nursing*	South Korea	Pre-post-test interventional study
***Rogers, 2011 *** [51]	Developing simulations in multiuser virtual environments to enhance healthcare education	*British Journal of Educational Technology*	Australia	Qualitative study
***Saab *et al*., 2021 *** [52]	Incorporating virtual reality in nurse education: a qualitative study of nursing students’ perspectives	*Nurse Education Today*	Ireland	Qualitative descriptive study
***Sara*** **et al*., 2021*** [53]	The effects of computer-based simulation game and virtual reality simulation in nursing students’ self-evaluated clinical reasoning skills	*CIN: Computers, Informatics, Nursing*	Finland	Pre-post-test interventional study
***Watari *et al*., 2020 *** [54]	The utility of virtual patient simulators for clinical reasoning education	*International Journal of Environmental Research and Public Health*	Japan	Pre-post-test interventional study
***Williams *et al*., 2020 *** [55]	Teaching interprofessional competencies using virtual simulation: a descriptive exploratory research study	*Nursing Education Today*	Canada	Pre-post-test descriptive qualitative study
***Yang and Oh, 2022 *** [56]	The effects of neonatal resuscitation gamification program using immersive virtual reality: a quasi-experimental study	*Nurse Education Today*	South Korea	Quasi-experimental study

### Use of VR interventions within simulation training for SA and DM

Seventeen studies used computer-based VR interventions [25, 26, 28, 29, 34, 35, 37, 41, 45–47, 49–51, 54, 55, 58], while 16 studies used HMD-VR [24, 27, 31–33, 36, 38–40, 42–44, 48, 52, 56, 57]. The computer-based interventions were predominantly screen based; however, three studies employed tablet- or mobile-based VR [35, 37, 45]. Sara et al. [53] used both computer-based and HMD VR interventions in their study [53]. Chen and Liou classified their application viewed from a smartphone as AR [30]. Nine studies compared VR-based interventions to traditional teaching or mannequin-based simulation methods [26, 35, 37, 39, 42, 43, 47, 49, 56] (Table 2) .

**Table 2:** Intervention

*Author, year of publication*	Aim of study	Terminology	Definition of terminology (as in study)	Commercial products used
***Adhikari *et al*., 2021 *** [57]	To investigate the impact of IVR sepsis game on preregistration nurses’ self-efficacy and their perceptions of the acceptability and applicability of IVR sepsis game as an adjunct to nursing simulation education	IVR (HMD)	Three-dimensional computer-based simulation that portrays a real-life situation in a computerized environment giving the user a sense of being present in the situation	NA
***Anbro *et al*., 2020 *** [24]	To evaluate changes in communication accuracy during an emergency medical simulation as a function of an interprofessional TeamSTEPPS® training	VR (HMD)	Provides an immersive audio/visual experience within a virtually simulated environment	• TeamSTEPPS® interprofessional training tool • HTC Vive VR Headsets with integrated Tobii eye-tracking sensors • SimMom®
***Atthill *et al*., 2021 *** [25]	To compare how student self-confidence and anxiety for engaging in CDM was impacted by a virtual asynchronous and face-to-face debriefing strategy after a VSG	VSG, virtual asynchronous debriefing (screen-based VR)	• VSG — the recreation of a human clinical scenario requiring the response of the learner in an evolving scenario, where they apply their nursing knowledge to determine and select critical nursing actions for a virtual patient • Virtual asynchronous debriefing — faculty-facilitated asynchronous debrief using 3D debriefing model over 48 h	NA
***Blanie *et al*., 2020 *** [26]	To compare simulation by gaming versus traditional teaching	SG (screen-based VR)	Video games were developed specifically with educational purposes. They can be computer based or use more immersive technologies such as virtual reality combined with head-mounted display	• Game developed in collaboration with LabForSIMS (simulation centre of Paris-Sud University), four nursing institutes, and a software designer Interaction Healthcare®, Levallois-Perret, France • Game developed — LabForGames Warning
***Bracq *et al*., 2021 *** [27]	To assess “Error recognition in a virtual operating room”, using a simulation scenario designed to improve situation awareness (awareness, subjective workload, anxiety, and user experience)	VR simulation (HMD)	Low-cost, realistic, easy to use, and easily configurable simulators using head-mounted displays (HMDs) which can be used to assist trainees acquire skills in a safe environment with low levels of anxiety	• HTC Vive System
***Carrard *et al*., 2020 *** [28]	To explore students’ perspective on the added value of a virtual patient (VP) simulation as part of a breaking bad news training in undergraduate medical education	VP (screen-based VR)	Computer-generated and manipulated graphical representation of a human (avatar) portraying patients in virtual environments used to train diverse medical competences such as making a diagnosis or therapeutic decisions	• Vizard 5.7 platform
***Casler *et al*., 2022 *** [29]	To compare the student debriefing experience in two methods of debriefing- automated software-generated performance feedback as the sole method of debriefing or self-review of software-generated feedback followed by an online, asynchronous, group discussion-board debriefing exercise	VS, faculty-guided asynchronous debriefing discussion (screen-based VR)	• VS — typically conducted outside of a traditional physical classroom and often involves a computer-based simulation using a virtual patient or avatar. They can also involve strategies such as virtual reality, mixed reality, and augmented reality • Faculty-guided asynchronous debriefing — debriefing strategy that combined self-review of software-generated feedback followed by an online, asynchronous, group discussion-board debriefing exercise facilitated by faculty	• i-Human (Kaplan Inc., Fort Lauderdale, FL, USA)
***Chen and Liou, 2022 *** [30]	To understand attitudes, perspectives, or beliefs about an AR-based assessment system for infant airway obstruction first aid training	AR (tablet- and computer-facilitated AR)	Combines computer-generated images, objects, information, or scenarios with the real-world environment and provides interactive experiences to users. Incorporates three features: a combination of real and virtual worlds, real-time interaction, and a requirement for a 3D space	• Unity (cross platform game engine) • Vuforia AR
***Cieslowski and Haas, 2023 *** [31]	Learner evaluation of VR experiences from student reflections and online survey	IVR (HMD)	A teaching modality that provides a three-dimensional platform which allows the end user to feel present and interact with the virtual world	• OMEN gaming laptops • Oculus Quest headsets
***Colonna*** **et al*., 2022*** [32]	To develop a novel trauma VR simulator, which would differentiate competency between participants with increasing levels of training and outcomes measured: time to correct treatments	TVRSim (HMD)	N/A	• Oculus Quest VR headset
***Du *et al*., 2022 *** [33]	To create and test a scenario-based, mixed-reality platform suitable for training NTS in battlefield first aid	MR (HMD)	A platform that can blend real-world objects of training relevance with VR reconstructions of operational contexts	• HTC Vive Pro 2.0 head-mounted device • HTC controller • Unreal Engine 4 (epic games)
***Fogg *et al*., 2020 *** [34]	To evaluate the clinical judgement skills of undergraduate baccalaureate nursing students using VR	VS (screen-based VR)	Computer-based recreation of reality that places users in an autonomous role controlling the environment	NA
***Hu*** **et al*., 2022*** [35]	To explore the effectiveness of a VR mobile game-based application for teaching disaster evacuation management to nursing students	Immersive VR-MGBA (screen-based VR — mobile device)	NA	NA
***Jayasundera *et al*., 2022 *** [36]	To evaluate the usefulness of VR simulation in reviewing pathophysiologic mechanisms of clinical decision-making	VR (HMD)	Generates a 360-degree experience to replicate a clinical situation via a head-mounted display (HMD)	• Oxford Medical Simulation • Head-mounted device
***Sahin Karaduman and Basak, 2023*** [37]	To compare the effects of virtual and human patient simulation methods on performance, simulation-based learning, anxiety, and self-confidence with clinical decision-making scores of nursing students	VPS (screen-based VR — large tablet simulator)	Computer-based clinical scenarios that are related to real life, where students can learn the necessary knowledge and practices for managing patient care. They generally use video of patients/actors or computer-generated human-like characters known as avatars	• BodyInteract® (Body Interact Inc., Austin, T, USA) • CAE Juno® (CAE Inc., Sarasota, FL, USA)
***Pardue*** **et al*., 2022*** [38]	To determine nursing students’ learning experiences when engaged in VR simulation and to explore learners’ problem-solving/clinical reasoning approaches after involvement in a VR simulation scenario	VR (HMD)	Three-dimensional environment using a headset that includes learner immersion or a sense of presence, visual feedback, and interactivity through user input or reactivity to the virtual platform	• Oxford Medical Simulation
***Kim*** **et al*., 2023*** [39]	To determine the impact of mixed simulation on learning how to provide nursing care for patients with arrhythmia	360° VR simulation followed by HFS (HMD)	VR involves the creation of three-dimensional type of content for users with a view in all directions. A 360° video (panoramic, spherical, or omnidirectional video/image) provides an immersive experience, allowing the user to explore a three-dimensional world built with 360° videos while they naturally change their viewpoints	• Insta360 Pro 2 (Arashi Vision Inc., Shenzhen, China) • Unity3D game engine (Unity Technologies, San Francisco, CA, USA) • YouTube • Oculus Quest 2 (Meta Platforms Inc., Cambridge, MA, USA) • SimMen
***Kiyozumi et al., 2022*** [40]	To create VR content for the Japan Prehospital Trauma Evaluation and Care programme and verify its educational effectiveness	VR (HMD)	N/A	• IDEALENS K4 (Idealens Technology Co., Ltd., Chengdu, Sichuan, China) • GoPro Max (GoPro Inc., San Mateo, CA, USA) • Adobe Premiere (Adobe Inc., Sa Jose, CA, USA) • VR content creation system (modified CREEK & RIVER scenario branching system; CREEK & RIVER Co., Ltd., Tokyo, Japan)
***Kleinheksel, 2014 *** [41]	A secondary data analysis of nursing students’ performance in the digital clinical experience	VP (screen-based VR)	Create an objective learning environment by presenting each student with an identical simulation scenario, allowing more uniform opportunity to assess skills and standardize patient actors	• Shadow Health Digital Standardized Patient™ (Digital Clinical Experience)
***Lee*** **et al*., 2022 *** [42]	To examine the effectiveness of mental-health nursing using VR simulation on knowledge acquisition, problem-solving ability, and learning satisfaction	360-degree VR (HMD)	Users can experience a higher sense of presence and engagement allowing them to be immersed in a virtual story	• Oculus Go — VR HMD • Gear 360 (360-degree camera)
***Lee *et al*., 2023 *** [43]	To develop a VR-based nursing education programme aimed at improving nursing students’ severity classification competency	VR (HMD)	Technology that maximizes user’s visual experience and immersion with the synthetic multi-needs of five senses. A representative, realistic media tool that enables users to have a more immersive experience of a given situation	• Oculus Quest 2
***Mahling *et al*., 2023 *** [44]	To assess the perceptions of a large cohort of students towards VR-based training and to identify the associations between these attitudes and individual factors, such as gender and age	VR (HMD)	Immerses the user in a completely virtualized world using special head-mounted devices. A special controller allows the user to interact with the VR	• Oxford Medical Simulation • Oculus Rift S (hand tracking device, Facebook Inc.) • Alienware m15R3 (high-performance laptop, Dell computers)
**Mascarenhas et al., 2023** [45]	To create an accessible and feasible methods of educating healthcare professionals about situational awareness in the hospital	Enhanced 360-degree video technology (screen-based VR — tablet)	Improves 360-degree video technology by measuring space and adding 360-degree photos to a mesh that it creates from the dimensions, allowing the user to independently navigate through the composite images	• Matterport™ (Sunnyvale, CA, USA)
***McCallum *et al*., 2011 *** [58]	To explore nursing students’ decision-making skills through the use of a 3D virtual environment such as second life	3D virtual environment (screen-based VR)	Residents of the virtual world can socialize, participate in individual and group activities, and create and trade virtual property in real time	• Second Life Clinical Simulation Laboratory
***Michelet *et al*., 2020 *** [46]	To focus on the impact of computer debriefing on learning acquisition and retention after a screen-based simulation training on neonatal resuscitation designed for midwifery students	Screen-based simulation Computer debriefing (screen-based VR)	NA	• Périnatsims (designed by Medusims)
***Middeke *et al*., 2018 *** [47]	To compare a serious game, the virtual accident and emergency department “EMERGE” to small-group problem-based-learning regarding student learning outcome on clinical reasoning in the short term	Serious games (screen-based VR)	Computer games designed for a serious purpose rather than pure entertainment	• EMERGE (PatientZero Games GmbH®)
***Mills*** **et al*., 2020*** [48]	To compare the simulation efficacy of a bespoke VR mass casualty incident triage training simulation against a comparable live simulation scenario	VR (HMD)	A simulated environment which uses digital sound and visual effects to create an authentic experience	• Purpose-built OZO VR camera (Nokia Pty Ltd., Espoo, Finland) • Oculus Rift (Oculus, Irvine, USA) • HTC Vive Headset (HTC Corporation, New Taipei City, Taiwan) • HTC Vive Controller
***Park *et al*., 2022 *** [49]	To compare the learning effects of a virtual simulation and high-fidelity simulation in a different order of presentation, with a focus on training for premature rupture of membranes in the field maternity nursing. To obtain evidence to support decision-making regarding the most effective way to utilize simulation strategies	VS (screen-based VR)	Provides a realistic world on the computer screen offering dynamic and consistent experiences in a safe, reproducible, accessible, and standardized clinical environment	• vSim® (Laerdal Medical)
***Rim and Shin, 2022 *** [50]	To develop a multiuser virtual simulation program for metacognition and to evaluate the students’ satisfaction, clinical judgement, and nursing competencies	VS (screen-based VR)	Clinical simulation offered on a computer, the Internet, or in a digital learning environment, including single or multiuser platforms	NA
***Rogers, 2011 *** [51]	To investigate how a simulation could be optimized in Second Life to encourage teamwork and collaborative problem-solving based on the habits, experiences, and perceptions of nursing students towards Second Life as a simulation platform	CBCS (screen-based VR)	Enables learners to interact with and manipulate information and representations of an environment and synchronously communicate with other people via a digital representation known as an avatar, regardless of their location	• Second Life (Linden Laboratories)
***Saab *et al*., 2021 *** [52]	To explore nursing students’ perspectives of incorporating VR in nurse education	VR (HMD)	A wide variety of computer-based applications commonly associated with immersive, highly visual, 3D characteristics to allow the participant to look about and navigate within a seemingly real or physical world	• Wireless VR headset • Headphones • Handheld controllers with haptic/vibrational feedback
***Sara*** **et al*., 2021*** [53]	To evaluate the effects of two different simulation games using a one-group pretest–posttest design	Simulation games (HMD & screen-based VR)	Artefacts (software) that replicate clinical reasoning processes in real-world situations. They can be used with different technologies, including mobile phones, tablets, computers, and virtual reality headsets	• HTC Vive Pro headset
***Watari *et al*., 2020 *** [54]	To clarify the effectiveness of virtual patient simulations for improving clinical reasoning skills among medical students and to compare improvements in knowledge or clinical reasoning skills relevant to specific clinical scenarios	VPS (screen-based VR)	NA	• Body Interact®, Coimbra, Portugal • TurningPoint® clicker, Tokyo, Japan
***Williams *et al*., 2020 *** [55]	To determine if VR supports the development of interprofessional competency knowledge	Virtual learning (screen-based VR)	Computer- or Internet-based learning environment that includes virtual worlds with avatars	• Clinispace® VR platform
***Yang and Oh, 2022 *** [56]	To examine the effects of a neonatal resuscitation gamification programme using immersive VR based on Keller’s ARCS model	IVR gamification program (HMD)	NA	• High-fidelity simulator (for simulation group) — Laerdal® Premature Anne

*NA* Not applicable, *IVR* Immersive virtual reality, *HMD* Head-mounted device, *VR* Virtual reality, *VSG* Virtual simulation game, *3D* three dimensional, *SG* Serious games, *VP* Virtual patient, *VS* Virtual simulation, *AR* Augmented reality, *TVRSim* Trauma virtual reality simulation, *MR* Mixed reality, *VR-MGBA* Virtual reality mobile game-based application, *HFS* High-fidelity simulation, *CBCS* Computer-based clinical simulation

### Participants and outcomes measured

Twenty-four articles looked at the role of VR for behavioural skills in nursing education [24–27, 29–31, 34, 35, 37–39, 41–43, 49–53, 55–58]. Ten articles looked at the role of the intervention for medical students [24, 28, 32, 33, 36, 40, 44, 45, 47, 54], 2 for paramedical education [40, 48], 1 for midwifery education [46], and 1 for healthcare assistant education [55]. Three papers studied the intervention in multiple groups of undergraduate healthcare professionals [24, 40, 55]. Twenty-nine articles studied the role of the intervention in training clinical judgement and related skills (i.e., problem-solving, decision-making, clinical reasoning) [25, 26, 30–44, 46–54, 56–58], and 6 articles looked at SA [24, 27, 28, 45, 46, 55]. One article looked at the effect of asynchronous debriefing on the virtual simulation experience [29]. The results and intervention-outcome effects of each study can be found in Table 3.

**Table 3:** Participants and outcomes

*Author, year of publication*	Participant group	Number of participants	Outcome(s) studied	Results
***Adhikari *et al*., 2021 *** [57]	Third-year nursing students	19	Decision-making	• There was a 26.1% increase in mean confidence with decision-making scores on the NASC-CDM post-intervention (*p* < 0.001) • There was a 23.4% decrease in anxiety with decision-making scores on NASC-CDM post-intervention (*p* < 0.001)
***Anbro *et al*., 2020 *** [24]	Third-year medical and nursing students	Third-year medical students = 49 Third-year nursing students = 56 Participants using HTC Vive Headset with eye tracking = 22	Communication accuracy, situational awareness	• Seventeen participants increased their frequency of correct verbal responses posttest — 5 looked at the speaker longer, 11 looked at the environment longer, and 1 did not change • Four participants did not increase their frequency of correct verbal responses posttest — two looked at the speaker longer, and two looked at the environment longer • One participant decreased their frequency of correct verbal responses posttest — looked at the speaker longer
***Atthill *et al*., 2021 *** [25]	Nursing students	Asynchronous debriefing = 32 Face-to-face debriefing = 32	Decision-making	• Both the asynchronous and face-to-face debriefing strategies showed an increase in self-confidence and decrease in anxiety with decision-making scores posttest • There was a significant change in post-test scores for all asynchronous debriefing dimensions. There was only a change in Dimension 2 of NASC-CDM for self-confidence and Dimension 3 of NASC-CDM for anxiety and self-confidence in face-to-face debriefing groups • The asynchronous debriefing strategy had a greater impact in reducing anxiety with relation to Dimension 1 of the NASC-CDM
***Blanie *et al*., 2020 *** [26]	Second-year nursing students	Simulation by gaming group = 73 Traditional teaching group = 73	Clinical reasoning	• There was no significant difference between groups in the script concordance test (SCT) scores immediately after and 1 month after the intervention (*p* = 0.43, *p* = 0.77) • There was no significant difference between groups in self-assessment of clinical reasoning knowledge after the intervention • Students in the simulation group expressed significantly more satisfaction towards the training session compared to the traditional teaching group (*p* = 0.001) • Students in the simulation group expressed more satisfaction towards the pedagogical tool compared to the traditional teaching group (*p* = 0.004) • Students in the simulation group perceived the session to be more engaging, with increased motivation levels (*p* = 0.003) • The global educational value (Would you recommend this training to students or colleagues?) was more positively significant in the simulation group (*p* = 0.002)
***Bracq *et al*., 2021 *** [27]	Scrub nursing students, expert scrub nurses	Scrub nursing students = 18 Expert scrub nurses = 8	Situational awareness	• Post first simulation, 3 students assessed their performance as poor, 10 as average, and 5 as good • The mean number of detected errors was higher in session 2 than session 1 (*p* < 0.001) • The mean number of reported non-errors was higher in session 2 than session 1 (*p* = 0.004) • Moderate risk detection rate was higher in session 2 than session 1 (*p* < 0.001) • Subjective workload was higher in session 1 than session 2 (*p* = 0.006) • Ease of use was higher in session 2 than session 1 (*p* = 0.019)
***Carrard *et al*., 2020 *** [28]	Fourth-year medical students	64	Situational awareness (verbal and nonverbal communication and behaviours, management of emotional reactions, pauses, and silences)	• Students highlighted the benefit of the simulation providing self-observation via a video recording enabling them to have a critical view of their behaviour • Some students noted that the simulation could be improved by indicating the “right” way of doing things (on-screen guidelines or examples)
***Casler *et al*., 2022 *** [29]	Nursing students	68	Effect of asynchronous debriefing discussion on virtual simulation experience	• Overall mean debriefing experience scale (DES) scores increased post-discussion from 66.3/100 to 72.0/100 • All 20 items of the DES showed an increase in post-discussion. However, 14 of the 20 items showed statistically significant differences • Fifty-one students described the combined debriefing strategy in positive terms and that the discussion-board debriefing exercises added value to their learning
***Chen and Liou, 2022 *** [30]	Nursing students	82	Clinical judgement, decision-making	• Students reported that the AR simulation inspired empathy, improved clinical judgement and decision-making, provided a stress-free learning environment, and improved the efficiency of distance and self-learning
***Cieslowski and Haas, 2023 *** [31]	Nursing students	110	Decision-making	• Students expressed that the scenario allowed them to critically think • Students noted that the VR scenario imparted greater confidence and comfort before approaching the scenario in real life
***Colonna*** **et al*., 2022*** [32]	Fourth-year medical students, general surgery residents, surgical faculty	Fourth-year medical students = 6 General surgery residents = 18 Flight nurses = 2 Acute care surgeons = 4 Surgical oncologist = 1	Decision-making	• Participants noted the simulation’s ability to develop clinical decision-making ability. The Simulation Experience Scale (SSES) score for the clinical reasoning section was 4.31 ± 0.20 (mean ± SD)
***Du *et al*., 2022 *** [33]	Fourth-year medical students	20	Decision-making	• Students’ knowledge acquisition scores after training were significantly higher than before training (*p* ≤ 0.001) • In the real-world scenario-based test students’ non-technical skills (NTS), scores were significantly higher after training compared to before training (*p* ≤ 0.001) • Total score obtained by students after training was significantly higher than before training (*p* ≤ 0.001) • There was no significant difference between scores of technical skills before and after training (*p* = 0.69)
***Fogg *et al*., 2020 *** [34]	Nursing students	234	Clinical judgement	• There were significant improvements in the Lasater Clinical Judgement Rubric (LCJR) scores after the simulation, compared to before (*p* = 0.000) • LCJR ratings were significantly improved in the fields of noticing (*p* = 0.000), interpreting (*p* = 0.002), responding (*p* = 0.001), and reflecting (*p* = 0.01) after the simulation, compared to before • The number of student attempts decreased significantly from the first case to the final case simulation (*p* = 0.000) — student attempts ranged from 1–13 on the first case to 1–5 on the last. Student scores in both cases ranged from 90 to 100%
***Hu*** **et al*., 2022*** [35]	Nursing students	Traditional teaching method = 80 Virtual reality mobile game-based application (VR-MGBA) group = 78	Decision-making	• Final test scores revealed significantly higher knowledge and decision-making retention in the game group compared to the traditional teaching group (*p* = 0.000) • The game-based group displayed significantly higher scores for course interest and classmate cooperation compared to the traditional lecture group (*p* < 0.05)
***Jayasundera *et al*., 2022 *** [36]	Third-year medical students	8	Decision-making	• The students demonstrated statistically significant improvements in their confidence with making appropriate and timely clinical decisions (*p* = 0.008), effectively managing a team (*p* = 0.031), and keeping patients updated (*p* = 0.031)
***Sahin Karaduman and Basak, 2023*** [37]	Third-year nursing students	Two virtual patient simulation group = 42 Virtual patient simulation and human patient simulation group = 42 Control (human patient simulation) = 42	Decision-making	• There were no statistically significant differences between groups in nursing confidence with clinical decision-making pre-test (*p* = 0.231) and posttest (*p* = 0.953) • There were no statistically significant differences between groups in nursing anxiety with clinical decision-making pre-test (*p* = 0.605) and posttest (*p* = 0.907) scores • There was a statistically significant difference between the pre-test and post-test nursing self-confidence and anxiety with decision-making scores in the virtual patient simulation group (*p* < 0.001) • There was a statistically significant difference between the pre-test and post-test nursing self-confidence and anxiety with decision-making scores in the virtual patient + human patient simulation group (*p* = 0.042) • There was a statistically significant difference between the pre-test and post-test nursing self-confidence and anxiety with decision-making scores in the control group (*p* < 0.001) • The performance scores should be a statistically significant difference in simulation groups (*p* < 0.001). The highest performance score value was obtained in the virtual patient simulation group, and the lowest was obtained by the control group
***Pardue*** **et al*., 2022*** [38]	Nursing students	19	Clinical judgement	• Students engaged in virtual reality simulation scenarios successfully described and demonstrated one or more phases of nursing clinical judgement (i.e. effective noticing, effective interpreting, effective responding, effective reflecting)
***Kim*** **et al*., 2023*** [39]	Nursing students	Intervention group = 25 Control group = 23	Decision-making	• There was a significant increase in post-test scores in the intervention group from pre-test (*p* < 0.001). The control group also had a statistically significant increase in post-test knowledge scores (*p* = 0.017). The difference between the groups was not statistically significant (*p* = 0.594) • The intervention group had significantly lower post-test anxiety with clinical decision-making scores than the control group (*p* = 0.031) • The intervention group had significantly higher decision-making confidence scores in “knowing and acting” (*p* = 0.025) and “seeking information from clinical instructors” (*p* = 0.049) than the control group • The intervention group reported good learning immersion in VR-based mixed simulations
***Kiyozumi et al., 2022*** [40]	Paramedical and medical students	Medical students = 5 Paramedical students = 9	Decision-making	• There was a statistically significant improvement of the number of clears in the scenarios between the first 5 min and the second 5 min (*p* = 0.0125) • There was a statistically significant improvement in the number of clears between the first 5 min and the third 5 min (*p* = 0.0045) • The difference between the number of clears in the second and third 5 min was not statistically significant (*p* = 0.0915)
***Kleinheksel, 2014 *** [41]	Nursing students	130	Clinical reasoning	• The most significant predictor for the implications for practice reflection score was critical items discovered (*p* < 0.001) • The number of red flag items (negatively correlated) was a significant predictor for the implications for practice reflection score (*p* = 0.031)
***Lee *et al*., 2022 *** [42]	Nursing students	VR group = 56 Control group = 48	Problem-solving	• Problem-solving scores increased in both groups after the intervention. However, the improvement in the VR group was statistically significant in the decision-making, solution applying, and evaluation reflection domains (*p* < 0.05), while there was no statistically significant improvement in the control group • The learning satisfaction in the VR group was significantly higher overall and for each examined item (*p* < 0.001)
***Lee *et al*., 2023 *** [43]	Fourth-year nursing students	Experimental group = 17 Control group = 17	Decision-making	• There was a statistically significant difference between the experimental and control group’s decision-making scores posttest (*p* < 0.001) • The post-test scores for confidence in performance was significantly different between the groups (*p* < 0.001)
***Mahling *et al*., 2023 *** [44]	Fourth-year medical students	129	Decision-making	• 91% of respondents agreed that VR was useful in conveying complex issues quickly • 80% of respondents agreed that VR should be used in examinations
***Mascarenhas et al., 2023*** [45]	N/A	510	Situational awareness	• 80% of learners “strongly agreed” or “agreed” that the intervention improved their situational awareness to identify hazards that can be present within the patient room
***McCallum *et al*., 2011 *** [58]	Nursing students	5	Decision-making	• Students learnt clinical decision-making from the experience
***Michelet *et al*., 2020 *** [46]	Fourth-year midwifery students	Debriefing group = 14 Control group = 14	Decision-making, situational awareness	• No significant difference between the groups with regard to knowledge acquisition and retention after intervention • A significant difference was observed in the non-technical skills assessment between the two groups for session 1 (*p* = 0.02), which remained higher in favour of the debriefing group in session 2 (*p* = 0.08)
***Middeke *et al*., 2018 *** [47]	Fifth-year medical students	Problem-based learning group (PBL) = 34 EMERGE group = 78	Clinical reasoning	• Students in the EMERGE group achieved significantly higher aggregate scores compared to the PBL group (*p* = 0.015) • Students in the EMERGE group achieved significantly higher aggregate scores than students in the PBL group across all four cases (*p* < 0.001 (1–3); *p* = 0.004 (4))
***Mills*** **et al*., 2020*** [48]	Paramedical students	29	Decision-making	• Average heart rate was significantly higher during the live simulation compares to the VR simulation (*p* < 0.001) • The live simulation took significantly longer to complete compared to the VR scenario (*p* < 0.001) • No significant differences were observed in total score of satisfaction and importance components of simulation design scale (*p* > 0.05)
***Park *et al*., 2022 *** [49]	Nursing students	Group 1 (virtual simulation (VS) followed by high fidelity simulation (HFS)) = 26 Group 2 (high-fidelity simulation followed by virtual simulation) = 26	Clinical reasoning	• After the first simulation, a significant difference was seen in clinical reasoning (*p* = 0.031) and problem-solving process (*p* = 0.006) with higher scores in the virtual simulation group than the high-fidelity simulation group • The group that received VS first and HFS second had higher scores for reflective thinking (*p* < 0.001) and self-confidence (*p* = 0.013)
***Rim and Shin, 2022 *** [50]	Nursing students	45	Clinical judgement	• Students rated their clinical judgement scores as higher post-session compared to pre-session • Nursing competency scores significantly increased after the programme (*p* < 0.001)
***Rogers, 2011 *** [51]	Nursing students	16	Problem-solving	• Students reflected that the simulation experience helped in construction of knowledge and development of problem-solving skills in a collaborative environment
***Saab *et al*., 2021 *** [52]	Third-year nursing students	26	Decision-making	N/A
***Sara*** **et al*., 2021*** [53]	Nursing students	40	Clinical reasoning	• Students evaluated their clinical reasoning skills as best in collecting information and worst in establishing goals in all three phases • There was a significant improvement in self-evaluated clinical reasoning skills from before the simulations to after the virtual reality simulation
***Watari *et al*., 2020 *** [54]	Fourth-year medical students	169	Decision-making	• Participants showed a significant increase in their average total post-test scores compared to pre-test (*p* < 0.001) • The rate of change between pre-test and post-test clinical reasoning answers was higher than knowledge answers (*p* < 0.008)
***Williams *et al*., 2020 *** [55]	Nursing, practical nursing, and healthcare assistant students	Nursing = 27 Practical nursing = 12 Healthcare assistant = 7	Situational awareness (role awareness, positions of power)	• Students felt that the simulation provided them with an opportunity to discuss role challenges with their teammates • The simulation highlighted that despite different roles and natural positions of power, all members of the team are equally important
***Yang and Oh, 2022 *** [56]	Nursing students	Virtual reality group = 29 Simulation group = 28 Control group = 26	Problem-solving, clinical reasoning	• There was no significant difference in nursing knowledge scores between the virtual reality group and the simulation group. However, there was a significant difference in the scores between the virtual reality group and the control group (*p* < 0.001) • The problem-solving ability score increased from pre-test to posttest in the virtual reality and simulation groups but decreased in the control groups. The VR group scores were significantly higher than both the other groups (*p* = 0.038) • The clinical reasoning ability score of the three groups increased from pre- to post-intervention. However, the virtual reality group did not significantly improve in comparison to the simulation and control groups • Self-confidence scores for all three groups increased post-intervention. The virtual reality group scores improved significantly compared to the other two groups • There were no significant differences in the anxiety scores between groups, although all groups anxiety scores were reduced post-intervention • There were no significant differences in learning motivation scores between the virtual reality group and the simulation group. However, the scores were higher compared to the control group

### Outcome measures used to assess impact of intervention on SA and DM

Eighteen studies used a validated method of assessment for outcome measurement [25, 27, 29, 31, 32, 34, 37–39, 41, 43, 46, 48–50, 53, 56, 57]. Of these, four studies used the Nursing Anxiety and Self Confidence with Clinical Decision-Making Scale (NASC-CDM) [25, 37, 39, 57], and three studies used the Lasater Clinical Judgement Rubric (LCJR) [34, 38, 50]. Details of other tools and methods of assessment can be found in Table 4.. The remaining 17 studies used a combination of project-developed Likert scale questionnaires, semi-structured interviews, one-to-one interviews, and focus groups to assess the outcomes [24, 26, 28, 30, 33, 35, 36, 40, 42, 44, 45, 47, 51, 52, 54, 55, 58].

**Table 4:** Educational theories and methods of assessment

*Author, year of publication*	Educational theory (if any)	Validated assessment tool	Unvalidated assessment tool	Method of assessment
***Adhikari *et al*., 2021 *** [57]	Gamification	X		• NASC-CDM scale • Qualitative data collection: Informal debrief and semi-structured interviews (focus group or one to one)
***Anbro *et al*., 2020 *** [24]	NA		X	• Predetermined verbal checkback opportunities • Eye gaze metrics for predetermined areas of interest
***Atthill *et al*., 2021 *** [25]	Experiential learning theory	X		• NASC-CDM
***Blanie *et al*., 2020 *** [26]	Experiential learning theory		X	• Script concordance tests (SCT) — case-based tests consisting of short scenarios for which each trainee must interpret newly formulated information against a baseline to modulate the final decision along a 5-point Likert scale • Self-assessment of perceived change in clinical reasoning process • Questionnaire to assess students’ perceived satisfaction, motivation towards learning, and effectiveness of the instructional design
***Bracq *et al*., 2021 *** [27]	3D model of debriefing	X	X	• Data regarding detected errors, time for detection, and trajectory patterns in the virtual OR for each participant • Situational Awareness Rating System (SART; Taylor, 1990) • The NASA Task Load Index (NASA-TLX; Hart and Staveland, 1998) • State-Trait Anxiety Inventory (STAI-Y; Marteau and Becker, 1992) • Collective debriefing • Questionnaire for attitudes and satisfaction post-simulation
***Carrard *et al*., 2020 *** [28]	N/A		X	• Focus groups
***Casler *et al*., 2022 *** [29]	Experiential learning theory, 3D model of debriefing	X		• Debriefing Experience Scale (DES) • Post-test survey — open-ended questions
***Chen and Liou, 2022 *** [30]	N/A		X	• Focus-group interviews (consisting of six 50-min sessions with 13–14 participants each)
***Cieslowski and Haas, 2023 *** [31]	Jefferies Simulation Theory	X	X	• Debriefing after each VR simulation session • Weekly student reflections • Simulation Effectiveness Tool-Modified (Leighton et al., 2018) online survey
***Colonna*** **et al*., 2022*** [32]	NA	X		• Satisfaction with Simulation Experience Scale (SSES) • Fast Form of Technology Acceptance Model (FF-TAM)
***Du *et al*., 2022 *** [33]	NA		X	• Knowledge acquisition regarding non-technical skills (NTS) form • Real-world scenario-based tests • Knowledge acquisition test
***Fogg *et al*., 2020 *** [34]	NA	X	X	• Lasater Clinical Judgement Rubric (LCJR) • Individual case scores and number of attempts
***Hu*** **et al*., 2022*** [35]	NA		X	• Twenty multiple-choice questions to test mastery of essential disaster evacuation management educational knowledge and decision-making abilities — administered as a pre-test, posttest, and final test • Instructional mode opinion survey
***Jayasundera *et al*., 2022 *** [36]	NA		X	• Pre-post-virtual reality simulation confidence ratings (17-item questionnaire)
***Sahin Karaduman and Basak, 2023*** [37]	Experiential learning cycle	X	X	• NASC-CDM • Simulation-Based Learning Evaluation Scale • Performance assessment forms • Student feedback forms
***Pardue*** **et al*., 2022*** [38]	NA	X	X	• Clinical judgement rubrics by Tanner and Lasater • Focus groups
***Kim*** **et al*., 2023*** [39]	NA	X	X	• 12-item multiple-choice written examination • NASC-CDM • Learning immersion in simulation instrument by Ko • The State Empathy Questionnaire by Shen • Usability instrument by Ingrassia et al
***Kiyozumi et al., 2022*** [40]	Experiential learning cycle		X	• Number of clears within the VR simulation program at 5-min intervals
***Kleinheksel, 2014 *** [41]	Constructivist theory (situated cognition and transformative learning theory)	X		• Reflection rating rubric (Cook (2010)) —clinical reasoning and implications for practice reflection
***Lee *et al*., 2022 *** [42]	Experiential learning theory		X	• Multiple-choice questionnaire (MCQ) for knowledge acquisition (18 item), problem—solving (30 items) • Likert scale questionnaire for learning satisfaction (nine items)
***Lee *et al*., 2023 *** [43]	5E circular learning model	X		• Severity classification competency measurement tool (Moon) • Confidence in Performance in Clinical Nursing Scale (Kim) • Clinical Decision-Making in Nursing Scale (Jenkins) • Simulation Design Scale (National League for Nursing revised by Yoo)
***Mahling *et al*., 2023 *** [44]	Experiential learning		X	• Questionnaire to assess learner perspectives on the curricular implementation of VR in emergency medicine (13 items, 5-point Likert scale)
***Mascarenhas et al., 2023*** [45]	NA		X	• Virtual room of errors entry form • Evaluation of the simulation experience (via Jotform)
***McCallum *et al*., 2011 *** [58]	NA		X	• Semi-structured, tape-recorded, one-to-one interview
***Michelet *et al*., 2020 *** [46]	NA	X		• Questionnaire based on International Liaison Committee on Resuscitation recommendations • Neonatal resuscitation performance evaluation (NRPE) system • Anaesthetists’ non-technical skills (ANTS) observation system
***Middeke *et al*., 2018 *** [47]	NA		X	• Formative key feature examination
***Mills*** **et al*., 2020*** [48]	NA	X	X	• Heart rate via Polar S610i watches and chest straps • National Aeronautics and Space Administration Task Load Index (NASA-TLX) • Simulation Design Scale • Triage card placement • Focus groups
***Park *et al*., 2022 *** [49]	NA	X		• Problem-Solving Process Behavior Survey (Park and Woo) • Nurses’ Clinical Reasoning Scale (NCRS) Korean Version • Reflective Learning Continuum (Peltier et al.) • National League for Nursing self-confidence and satisfaction with practicum scales
***Rim and Shin, 2022 *** [50]	Experiential learning theory	X		• Lasater Clinical Judgement Rubric • Korean Nurses’ Core Competency Scale
***Rogers, 2011 *** [51]	Constructivist learning theory		X	• Individual interviews
***Saab *et al*., 2021 *** [52]	N/A		X	• Sociodemographic questionnaire • Face-to-face individual/focus-group interviews
***Sara*** **et al*., 2021*** [53]	Gamification	X		• Clinical Reasoning Skills scale (CRS) (Levett-Jones et al.) • Simulation metrics
***Watari *et al*., 2020 *** [54]	N/A		X	• 20 multiple-choice questions — 10 knowledge and 10 clinical reasoning based. Administered before and after the intervention
***Williams *et al*., 2020 *** [55]	NLN/Jefferies Simulation Framework		X	• Semi-structured questionnaire
***Yang and Oh, 2022 *** [56]	Keller’s ARCS model	X		• Neonatal resuscitation nursing knowledge measurement tool standardized • Problem-solving ability by adult’s tool • Korean version of Nurses Clinical Reasoning Scale • Self confidence in performing neonatal resuscitation tool standardized • State-Trait Anxiety Inventory (STAI) translated in Korean • Learning Motivation Test Paper (Son and Keller (2001))

*NA* Not applicable, *NASC-CDM* Nursing anxiety and self-confidence with clinical decision-making, *3D* three dimensional, *ARCS* Attention, relevance, confidence, and satisfaction

### Educational theories underpinning VR simulation training for SA and DM

Seventeen articles described educational learning theories [59] which would guide the technique of learning with the intervention [25–27, 29, 37, 40–44, 50, 51, 53, 55–57, 60]. Eight papers used experiential learning theory [25, 26, 29, 37, 40, 42, 44, 50]; two papers used constructivist learning theory [41, 51], two papers used gamification [64] [53, 57] — a theory used to describe the use of game design elements in non-game contexts, rooted in behaviourism (see Wang et al. for further description [61]); two papers used the 3D model of debriefing [27, 29]; two papers used Jeffries Simulation Theory [55, 60]; one paper used the 5E circular learning model [43]; and one paper used Keller’s attention, relevance, confidence, and satisfaction (ARCS) model [56]. Eighteen articles did not report the use of educational theories to guide the learning techniques using the intervention [24, 27, 28, 30–36, 38, 39, 45–49, 52–55, 58]. Details of the learning theories used in each study can be found in Table 4. (for further information on learning theories, see Taylor and Hamdy [59]).

## Discussion

To the best of our knowledge, this is the first scoping review that provides a comprehensive synthesis of evidence surrounding the use of VR for the training of SA and DM skills for healthcare education. VR is a revolutionary technology that has the potential to remodel the landscape of healthcare education. As a result, it has attracted significant academic interest, with educators and academics exploring optimal contexts and methods for the use of VR in healthcare education. VR poses the advantages of accessible training “on demand” either in its immersive or non-immersive forms [5, 62].

### VR-based terminology

Within this review, VR was defined as technology that provides the user with a three-dimensional (3D) digital environment while allowing them to interact with its elements [22]. VR can be broadly categorized as immersive (consisting of HMDs, allowing complete involvement within the task environment) and non-immersive (consisting of screen-based displays which can be manually navigated by the user) [63, 64].

While studies were excluded if they had interventions that were not considered 3D or interactive, included studies were found to have high levels of disparity within their definitions of VR interventions. When categorizing studies based on use of immersive and non-immersive interventions, it was found that 16 studies employed the use of HMD-based VR, while the other 17 used screen-based non-immersive modalities. This disparity in terms of definitions of what constitutes VR has been described previously with Kardong-Edgren et al., calling for unifying definitions of VR, suggesting elements such as level of immersion considering characteristics of presence, based on the senses of the learners which various technologies are designed to deceive, are reported [7].

A recent review found that subjectively, learners report enjoying training with HMD, and advances in technology now result in fewer adverse effects such as “cybersickness” [65]. It is likely that the higher numbers of non-immersive VR studies in our review relate to the fact that SA and DM can be represented easily using non-immersive technologies, requiring less specialized equipment and are cheaper than immersive-VR options [66]. Cant and Ryan’s recent mapping review describes the range of virtual environments and technologies available for use within nursing education, either paid-for subscription options or open-access options using various technologies [67].

Winn [68] described learning in artificial environments to be influenced by both the adaptation of the learner to the environment, and the environment to the learner [68], highlighting the role of immersion in the learning experience. It is imperative that the right form of simulator is utilized to match the desired learning outcomes; however, it is noted that this is a complex and challenging process. Goodwin and Nestel describe the affordances and limitations of different simulation modalities, in particular associated cost benefits and flexibility/accessibility associated with computer-based VR simulations and the potential of improved psychological safety with solo use HMD-VR simulations [69]. This highlights the need for educators to consider the advantages and disadvantages posed by the level of immersion within given contexts, particularly with the increasing availability of XR/AR technologies where the user can interact with parts of a real-world environment with virtual overlays. Shin et al. recently conducted an integrative review into the use of virtual simulation in nursing which described virtual-specific characteristics that educators need to consider alongside general simulation characteristics when designing VR educational strategies [70]. These included instructor competency, mode of representation, participant role, interaction, type of platform, theoretical framework, and virtual ethics [70]. Without having clear descriptions and definitions within the included studies, it is challenging to draw firm robust conclusions. Our findings support those of Nuha et al. who reviewed the distance simulation literature which found an absence of standardization of terminology within that field which made the review challenging [71].

### Outcome measures to assess SA and DM within VR simulation

It was encouraging to see the use of validated outcome measures for SA and DM across 18 of the 35 included studies. The Kirkpatrick training model is a standardized framework to objectively evaluate the outcomes of training interventions at different levels [72]. This considers four levels of outcome evaluation, namely reaction of the learner, learning or skills acquired, behaviour or performance measures, and organizational outcomes [72]. While the included studies reported outcomes for learner reaction and acquired skills, there were no reports of behaviour or organizational outcomes.

Evaluating higher levels of the Kirkpatrick model in the context of SA and DM would pose the challenges of (1) reliable outcome measures to assess the changes in learner behaviour after SA and DM skill acquisition and (2) evaluating the effect of such skills on patient care at an organizational level. However, Kardong-Edgren [72] highlighted the importance and need for the evaluation of such long-term outcomes in simulation research, to better justify the introduction of such modalities into healthcare education [73]. Our findings align with a 2024 umbrella review of the in-person simulation literature where 83% of studies focussed on level 2 outcomes [74]. Our findings, which is most of the studies are at the lower end of the Kirkpatrick framework, are not surprising given the relative infancy of the introduction of these technologies within this particular area of healthcare education and identify future research opportunities focussing on how this can be transferred into higher level outcomes [71]. A recent meta-analysis of VR in nursing education suggested that VR was more effective than control conditions in knowledge improvement however found no difference when assessing skills, satisfaction, or confidence [75].

### Educational theories underpinning the use of VR in healthcare education

Only 13 of the included studies reference specific learning theories; the majority is describing experiential learning while the others describing constructivist, gamification, Keller’s ARCS model, and the 5E circular learning model (Table 4.). This meant 22 of the included studies did not describe the utilization of learning theories when delivering the VR simulation training intervention.

All educational interventions are complex interventions [76], and therefore, a key aspect of their design process should be the use of an underpinning theoretical framework, meaning at the design stage there should be theoretically driven hypothesis of why the intervention should work in a particular context; this understanding should be evidenced in any publication describing the use or development of a teaching intervention. To ensure instructional design for novel educational tools such as VR, the underpinning learning theories should be well described to better suit the desired learning outcomes of the simulation exercise [77]. In accordance with instructional design models, it is imperative that methods of instruction are clearly researched and outlined during the development phase of an intervention to ensure effective design and implementation [78]. Application of education theories not only enhance the efficacy of the training but also provide a framework for evaluation to enhance understanding of *how* such interventions work and how they could be revised to improve their efficacy further [59].

### Strengths and limitations

Scoping reviews are a useful tool to determine the scope and coverage of published literature on a given topic, particularly in emerging disciplines where the number of studies that are available restricts the possibility of systematic reviews [79]. A significant strength of our review is its adherence to the robust framework described by Arksey and O’Malley and reporting as per the PRISMA-ScR guidelines [21, 23] identifying 35 studies for inclusion.

Our review is not without limitations. Our review was limited to English language articles, and our search therefore may have missed articles from other languages, introducing potential bias into the search results. A significant limitation to this review was the variability in definitions of our intervention of interest, VR, within included studies. Confusion regarding terminology within this field has been noted previously, with the understanding of the components of VR being variable [22]; this may have impacted the ability to identify relevant studies via our search terms. However, to standardize the intervention for the purpose of this review, we used the definition by Abbas et al., to include studies assessing SA and DM training using a wide range of “virtual” interventions encompassed by VR [22]. The broad definition of the virtual environment in use allowed us to evaluate the various virtual simulation techniques currently used to train SA and DM skills.

Additionally, there was a lack of standardization of the definitions of SA and DM within the included studies, with some studies using “clinical judgement” and “decision-making” interchangeably. While the reviewers attempted to be systematic in the process, this may have led to the potential exclusion of some relevant research. Another potential limitation was the extraction of data by a single researcher. We also note that our inclusion criteria focussed on undergraduate programmes only and recognize that many healthcare professions programmes across the world are classified as graduate education programmes limiting the generalizability of this review in these settings. Scoping reviews are designed to map the territory on a given subject, and as such do not routinely assess the risk of bias or methodological rigour of the included studies which may limit the applicability of the results [80].

### Future work

Our scoping review was able to identify multiple future research priorities within the field of simulation-based education research. Firstly, the lack of standardization of VR-based intervention terminology and outcomes such as SA and DM call for consensus on standard definitions and reporting measures for educational research in this field. This would allow for more homogenous reporting of simulation-based studies.

Secondly, we recommend additional research comparing immersive and non-immersive VR interventions for training of behavioural skills, to ascertain the most optimal approach. Lastly, we recommend research to evaluate long-term impacts of VR-based training on learner behaviour and patient experience to strengthen the evidence surrounding VR-based modalities in the training of behavioural skills.

## Conclusion

This scoping review has mapped how VR is being used across the spectrum of health professionals’ education to teach and assess SA + DM. We recommend the development of standardized reporting guidelines for VR studies within healthcare education to ensure increased quality of studies within this area and for clear descriptions of level of immersion and presence to be described. We would encourage authors to adhere to the definitions and terminology as per the latest edition of the Healthcare Simulation Dictionary to enhance the standardization of reporting allowing readers to fully understand what technologies were used and how they are implemented [3]. Included studies reported a variety of outcome measures ranging from validated SA + DM scales to Likert scale questionnaires and interviews. Around half of included studies reported clear educational theories when describing their VR interventions. We would encourage future VR researchers to describe the educational theories which apply when implementing VR interventions.

## Data Availability

No datasets were generated or analysed during the current study.
